# DNMT1 overexpression predicting gastric carcinogenesis, subsequent progression and prognosis: a meta and bioinformatic analysis

**DOI:** 10.18632/oncotarget.21480

**Published:** 2017-10-04

**Authors:** Tianmiao Ma, Hao Li, Mingjun Sun, Yuan Yuan, Li-Ping Sun

**Affiliations:** ^1^ Tumor Etiology and Screening Department of Cancer Institute and General Surgery, The First Affiliated Hospital of China Medical University, Liaoning Provincial Education Department, Key Laboratory of Cancer Etiology and Prevention, China Medical University, Shenyang, Liaoning, China; ^2^ Digestive Department, The First Affiliated Hospital of China Medical University, Shenyang, Liaoning, China

**Keywords:** DNMT1, gastric cancer, meta-analysis, bioinformatic analysis

## Abstract

DNMT1 is important in maintaining DNA methylation, and participates in the oncogenesis via up- or down-regulation leading to hyper-methylation or hypo-methylation. In the meta and bioinformatic analysis, we found that DNMT1 expression was higher in gastric cancer, compared with normal (*p* < 0.00001), para-cancerous (*p* = 0.0004) and dysplasia (*p* < 0.00001) tissues. DNMT1 up-regulation was associated with gender (OR = 2.27, *p* = 0.006), differentiation (OR = 0.21, *p* = 0.01) and TNM stage (OR = 0.31, *p* = 0.0005). Through TCGA database, DNMT1 overexpression increased gastric cancer risk, but unrelated with clinicopathological parameters and prognosis. Kaplan-Meier plotter showed, an increasing expression of DNMT1 was positive for overall survival rates of patients with stage III and IV (*P* = 0.044; *P* = 0.047), N2 and N1-3 phases of lymph node metastasis (*P* = 0.023; *P* = 0.032), as well as those with or without distant metastasis (*P* = 0.0052; *P* = 0.021). For DNMT1 negative patients, the progression-free survival rates was better in patients with Her2+ or Her2- than positive ones (*P* = 0.00015; *P* = 0.031). Besides, surgery alone was effective for the overall survival rates in patients with DNMT1 high expression (*P* = 0.035), while 5-Fu was useful for those with low expression (*P* < 0.05). In conclusion, these findings provided evidence that DNMT1 expression might be employed as a potential marker to indicate gastric carcinogenesis and subsequent progression, even prognosis.

## INTRODUCTION

DNA methylation is the most common epigenetic modification in the mammalian genome [1], involved in several processes during mammalian development including gene expression regulation, genomic imprinting, and X-chromosome inactivation [2, 3]. Aberrant methylation patterns can be found in human tumors and many abnormalities [4]. DNA methylation is catalyzed by a family of DNA methyltransferases (DNMTs) [5]. It is well known that eukaryotes contain three classes of DNMTs (Dnmt1, Dnmt2, Dnmt3a /Dnmt3b). DNMT1 is a maintenance methylase, which is the most important in the whole process of DNA rmethylation, whereas DNMT3a/DNMT3b is involved in de novo methylation, and DNMT2 is involved in tRNA methylation [6].

DNMT1, the first discovered member of DNMTs [7], which locates in human chromosome 19p13.2, mainly works in maintaining the normal methylation in DNA replication [8, 9]. In S phase of cell division, DNMT1 is associated with DNA replication sites maintaining the methylation pattern in the newly synthesized strand, which is essential for epigenetic inheritance. And during G2 and M phases, DNMT1 maintains DNA methylation of replication independently to establish the methylation pattern in development [10, 11]. DNMT1 participates in the oncogenesis via up- or down-regulation leading to hyper-methylation or hypo-methylation, respectively [12]. Overexpression of DNMT1 has been detected in several kinds of human cancers. Chen CL [13] reported that DNMT1 mRNA overexpressed in primary and recurrent epithelial ovarian carcinoma. Zhu YM [14] demonstrated that dysregulated expression of DNMT1 in colorectal cancer. Peng DF [15] indicated that increased DNMT1 protein expression was present in multistage pancreatic carcinogenesis from the precancerous stage to malignant progression. And Feng Y [16] reported DNMT1 might play an important role in the early process of lung cancer.

Although some investigators found that the level of DNMT1 expression increased in GC tissues compared with adjacent mucosa or distal normal tissues [17–28], and many reports showed the relationship between DNMT1 expression and different clinicopathological significances in gastric carcinogenesis [17–19, 21, 24, 26–29], but the results were controversial. In addition, the role of DNMT1 in the prognosis of GC was still ambiguous. Therefore, it is necessary to identify eligible studies and perform a meta and bioinformatic analysis to evaluate the carcinogenesis progression and prognosis value of DNMT1 in GC patients.

## RESULTS

### Characteristics of eligible studies

As shown in Table 1, a total of 14 articles on the relationship between DNMT1 expression and risk, clinicopathological parameters as well as prognosis of GC were retrieved for our meta-analysis by immunohistochemistry in PubMed, Web of Science, BIOSIS and CNKI [17–30]. 7 articles contained studies with normal gastric mucosa [17, 18, 20, 21, 24, 26, 27], 2 articles contained studies with gastric precancerous lesions-dysplasia [26, 27] and 7 contained studies with paired adjacent mucosa [19, 20, 22–25, 28]. 9 articles showed the differences between DNMT1 expression and clinicopathological characteristics of GC, including age, gender, tumor location, tumor size, differentiation, depth of invasion, TNM staging, Lauren's classification, lymph node metastasis and vascular metastasis [17, 20, 21, 24, 26–30]. Additionally, the prognostic significance was analyzed in 3 articles [17, 19, 30].

**Table 1:** Basic characteristics of eligible studies

Study	Year	Country	Ethnicity	Antibody company	No. of Cases (M+/M–)	No. of Control (M+/M–)	Risk to cancer	Risk to aggress	Outcome	Quality
Gao Y [22]	2016	China	Asian	——	90/30	7/113	Up			6
F.Ksiaa [17]	2015	Tunisia	African	DakoCytomation	24/23	0/47	Up	Neg	Neg	7
Liang YY [24]	2015	China	Asian	BIOSS	43/17	59/61	Up	Neg		8
F.Ksiaa [18]	2014	Tunisia	African	DakoCytomation	20/23	0/43	Up			6
Cao XY [19]	2014	China	Asian	Santa Cruz	51/34	32/53	Up	Neg	Neg	7
Lu YO [23]	2013	China	Asian	——	15/5	6/14	Up			7
Jiang XJ [25]	2012	China	Asian	——	56/12	10/58	Up			8
Mutze K [30]	2011	Germany	European	Santa Cruz	105/22	0/0	Up	Pos	Pos	6
Sun N [26]	2011	China	Asian	BIOSS	48/14	36/47	Up	Neg		8
Han J [29]	2010	China	Asian	Abcam	55/40	0/0	Up			6
Liu B [27]	2009	China	Asian	ALEXIS	49/21	14/46	Up	Pos		7
Ding WJ [20]	2008	China	Asian	Santa Cruz	31/7	19/57	Up	Neg		7
Liu T [28]	2007	China	Asian	Santa Cruz	32/2	29/5	Up			7
Etoh T [21]	2004	Japan	Asian	Santa Cruz	97/37	0/134	Up	Pos		6

### Association between DNMT1 expression and GC risk

**Figure 1:**
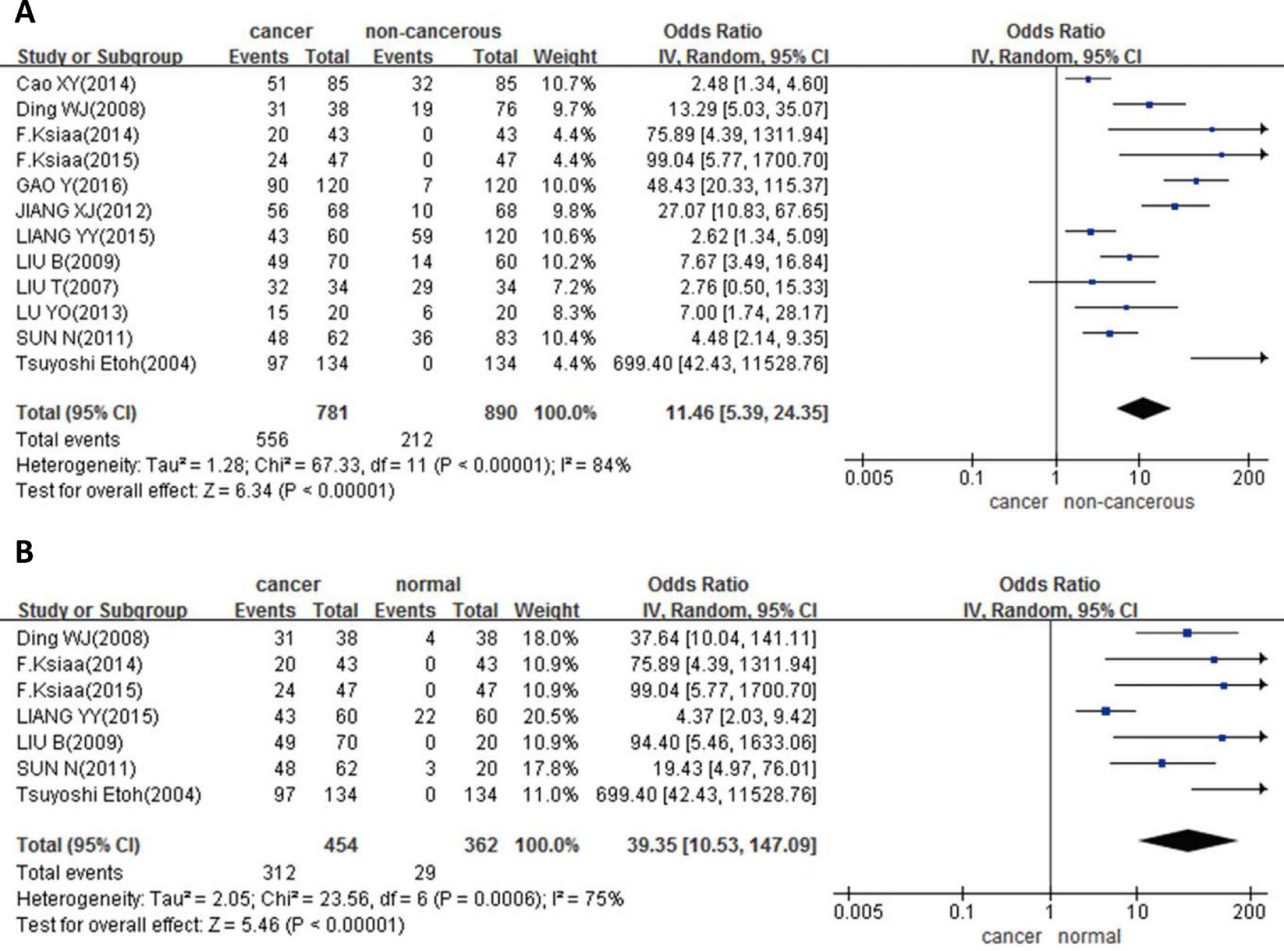

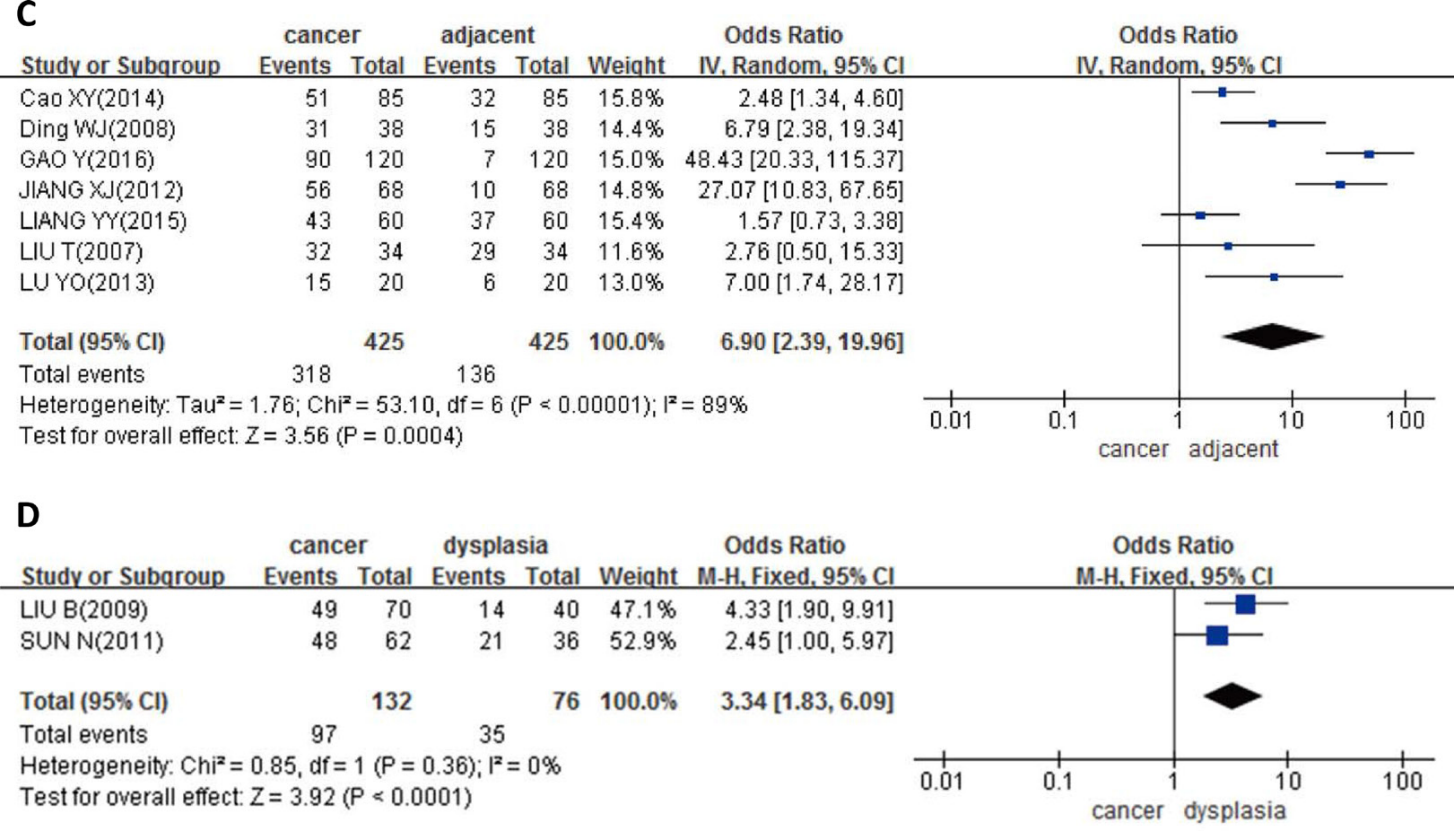
Forest plot for the relationship between DNMT1 expression and GC risk in different subgroups (**A**) Gastric carcinogenesis (cancer vs. non-cancerous); (**B**) gastric carcinogenesis (cancer vs. normal); (**C**) gastric carcinogenesis (cancer vs. adjacent); (**D**) gastric carcinogenesis (cancer vs. dysplasia).

In 12 studies with 781 cancers and 890 controls, we analyzed the DNMT1 different expression between GC and non-cancerous mucosa. The results showed, the DNMT1 expression was higher in GC than that in non-cancerous mucosa (*p* < 0.00001, Figure 1A). And in 7 articles with 454 cancers and 362 normal tissues, we observed the same trend in cancer compared with normal tissues (*P* < 0.00001, Figure 1B). When compared with paired adjacent cancerous samples, DNMT1 expression was also observed up-regulation in cancer in 7 studies with 425 cancers and 425 para-cancerous (*p* = 0.0004, Figure 1C). In addition, we also found higher DNMT1 expression in GC tissues than that in dysplasia tissues in 2 studies with 132 cancers and 76 dysplasia (*p* < 0.00001, Figure 1D).

### Association between DNMT1 expression and clinicopathological parameters of GC

**Figure 2:**
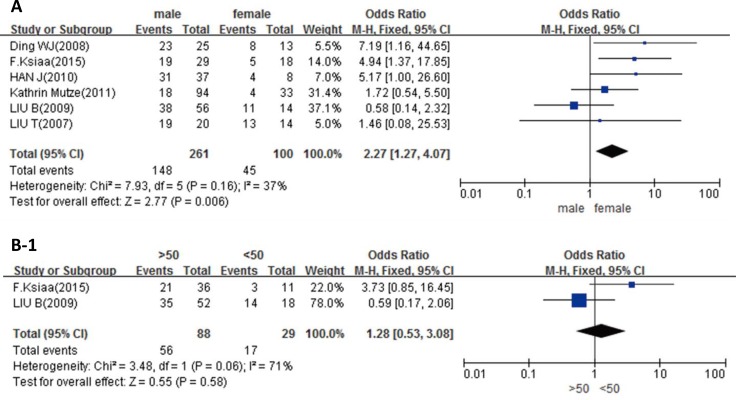

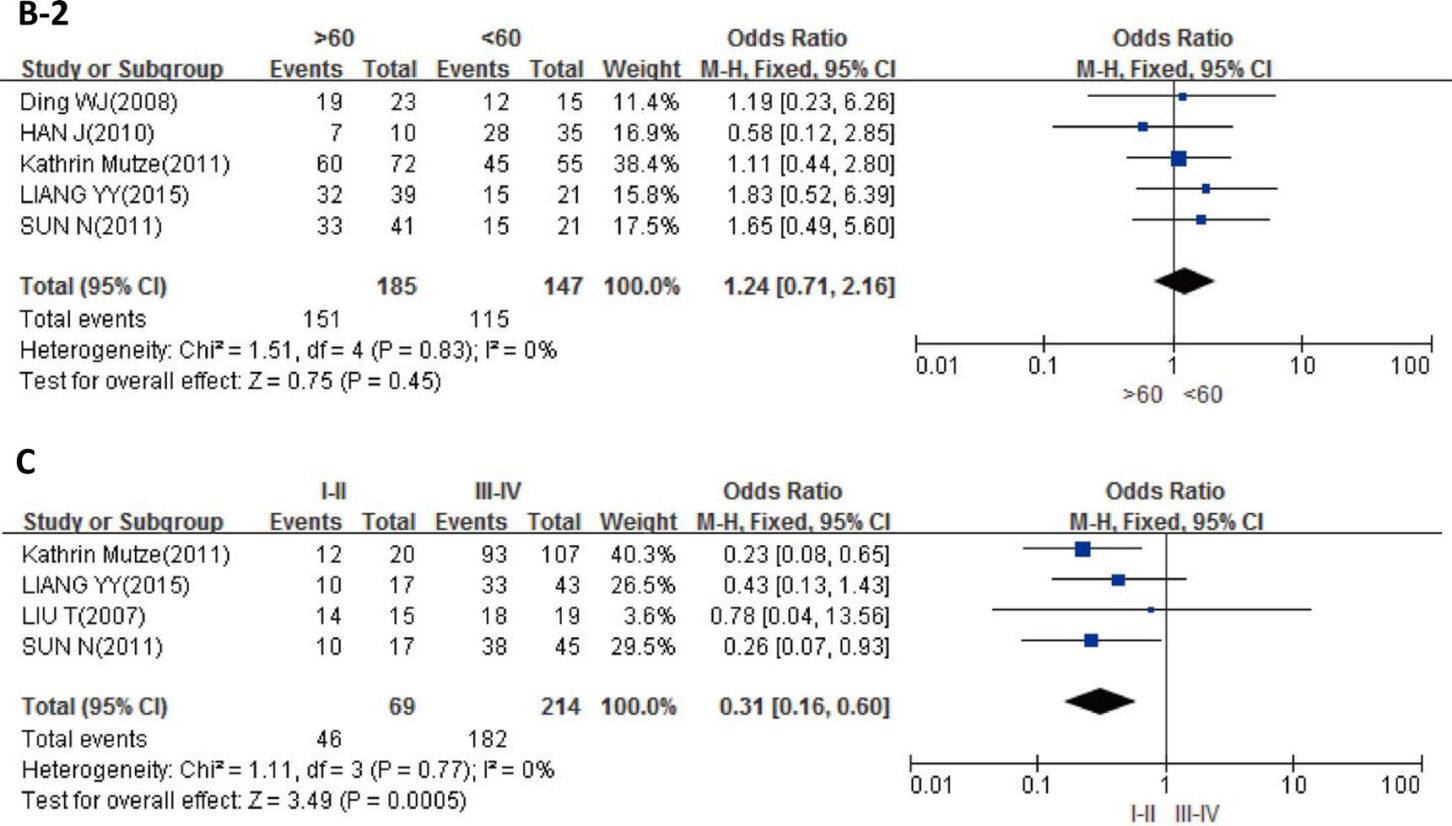

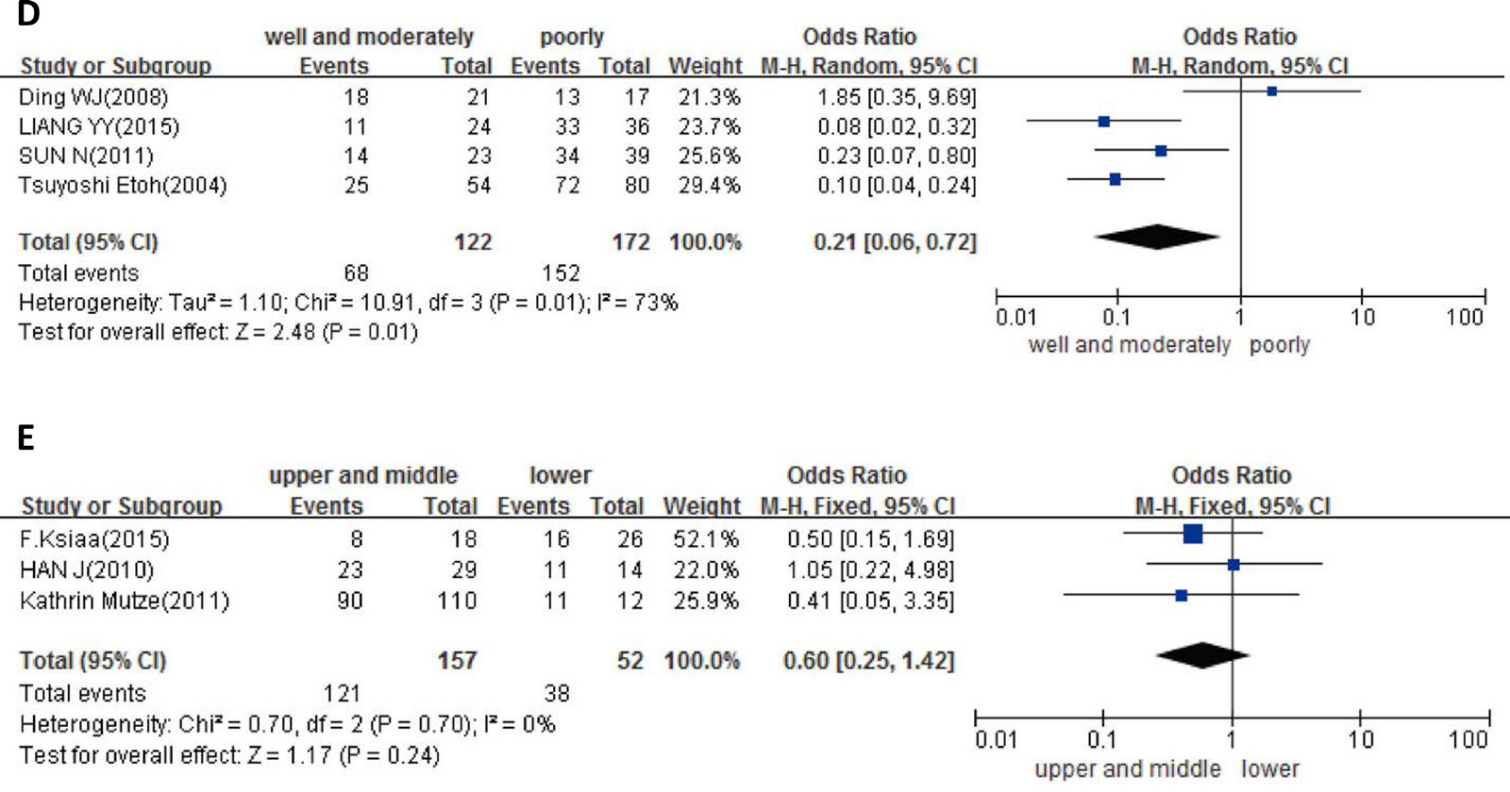

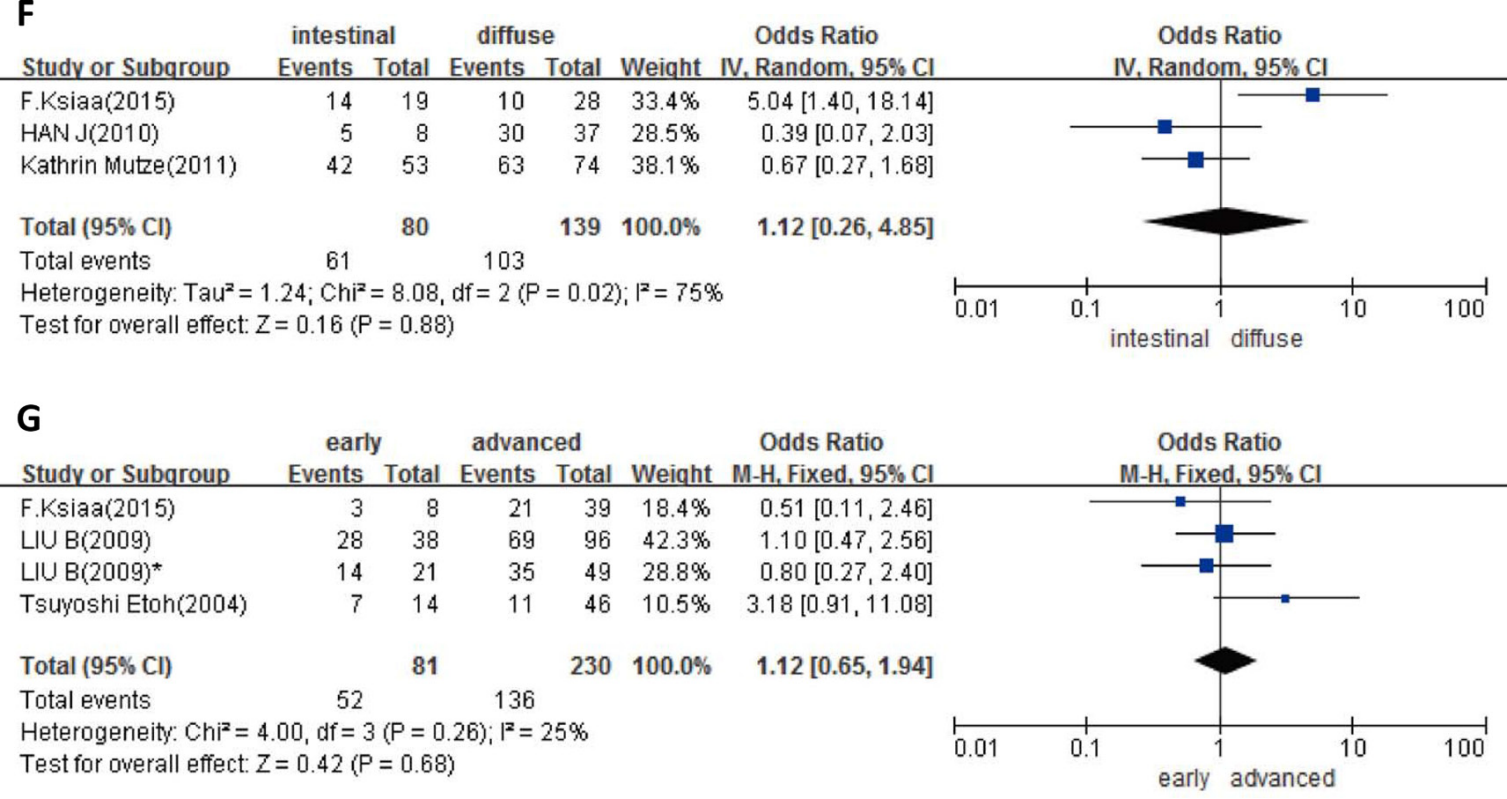

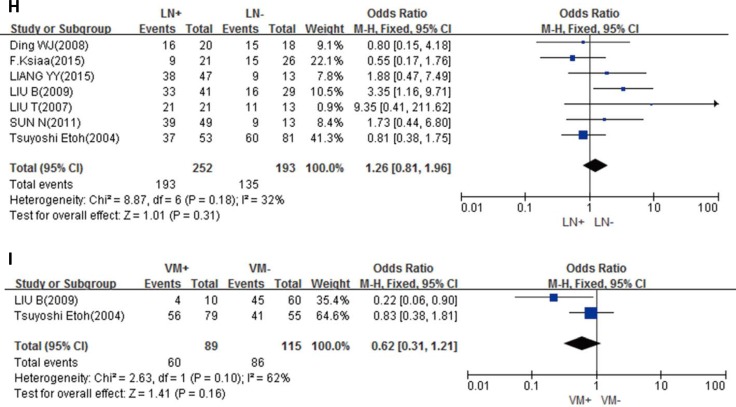
Forest plot for the relationship between DNMT1 expression and clinicopatholoiocal parameters of GC (**A**) Correlation between sex and DNMT1 expression (male vs. female); (**B-1**) correlation between age and DNMT1 expression (> 50 vs. < 50); (**B-2**) correlation between age and DNMT1 expression (> 60 vs. < 60); (**C**) correlation between TNM staging and DNMT1 expression (I-II vs. III-IV); (**D**) correlation between differentiation and DNMT1 expression (well and moderately vs. poorly); (**E**) correlation between tumor location and DNMT1 expression (upper and middle vs. lower); (**F**) correlation between Lauren's classification and DNMT1 expression (intestinal type vs. diffuse type); (**G**) correlation between depth of tumor and DNMT1 expression (early vs. advanced); (**H**) correlation between lymph node metastasis (LN) and DNMT1 expression (LN+ vs. LN–); (**I**) correlation between vascular metastasis (VM) and DNMT1 expression (VM+ vs. VM-).

As Figure 2A showed, in GC patients, the higher expression of DNMT1 was in males than that in females (*p* = 0.006), while no significant difference between age groups, no matter the criteria of distinction was 50 or 60 years old (*p* > 0.05, Figure 2B-1, 2B-2). Higher expression of DNMT1 was shown in Stage III-IV than Stage I-II (*p* = 0.0005, Figure 2C). In addition, DNMT1 was more expressed in poorly differentiated carcinoma than in well or moderately differentiated carcinoma (*p* = 0.01, Figure 2D). However, DNMT1 expression was not related to tumor location, Laruen's classification, depth of invasion, lymph node metastasis or vascular metastasis of GC (*p* > 0.05, Figure 2E–2I).

### Heterogeneity analysis

We did a subgroup analysis based on race. For African, *p* = 0.90, I^2^ = 0%; for Asian, *p* < 0.00001, I^2^ = 85%. The results indicated that race might be the main source of heterogeneity.

### Publication bias and sensitivity analysis

**Figure 3:**
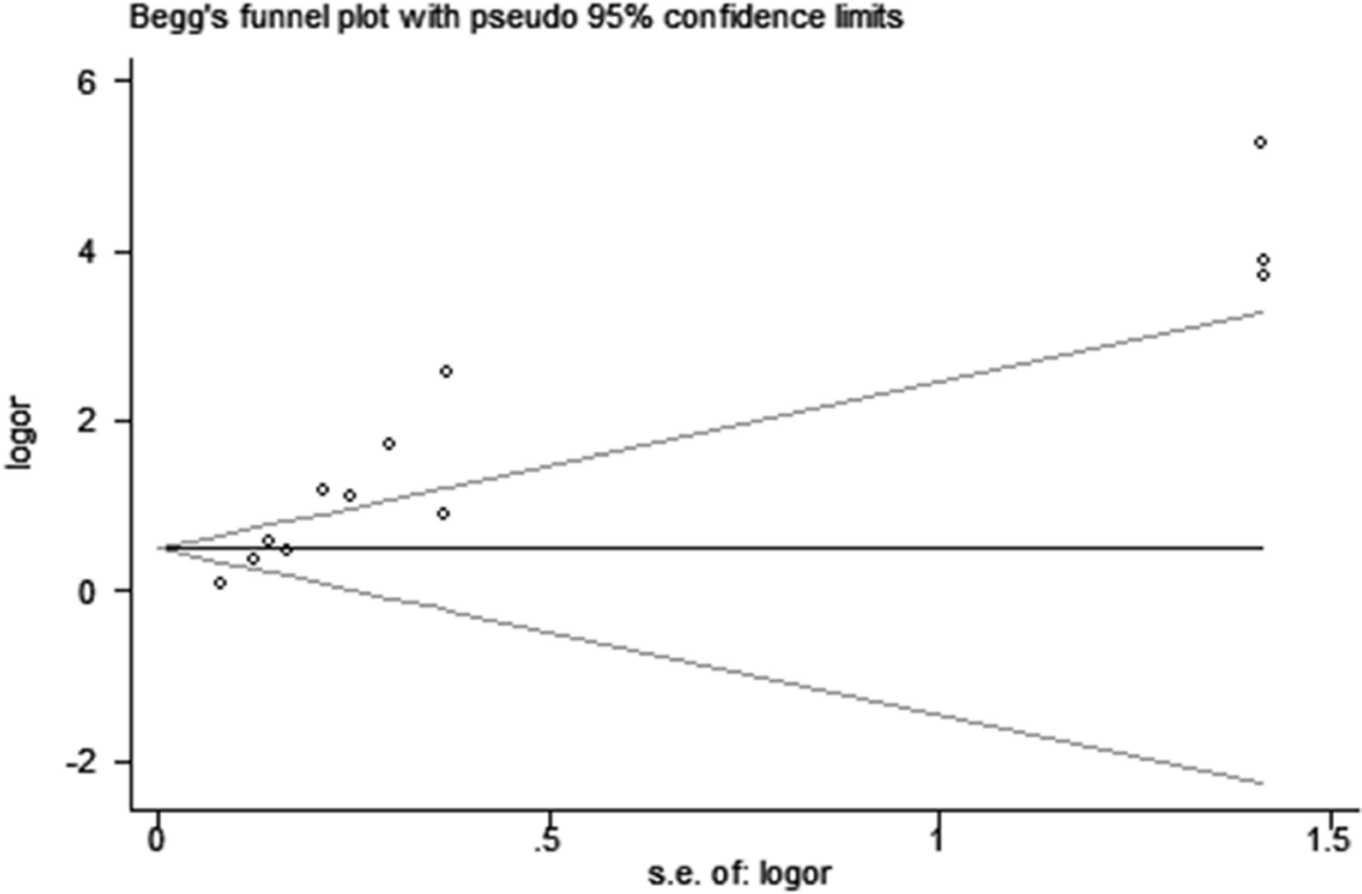
The funnel plot graphics of publication bias The bias was analyzed about risk degrees of DNMT1 expression in gastric mucosa for gastric carcinogenesis.

**Figure 4:**
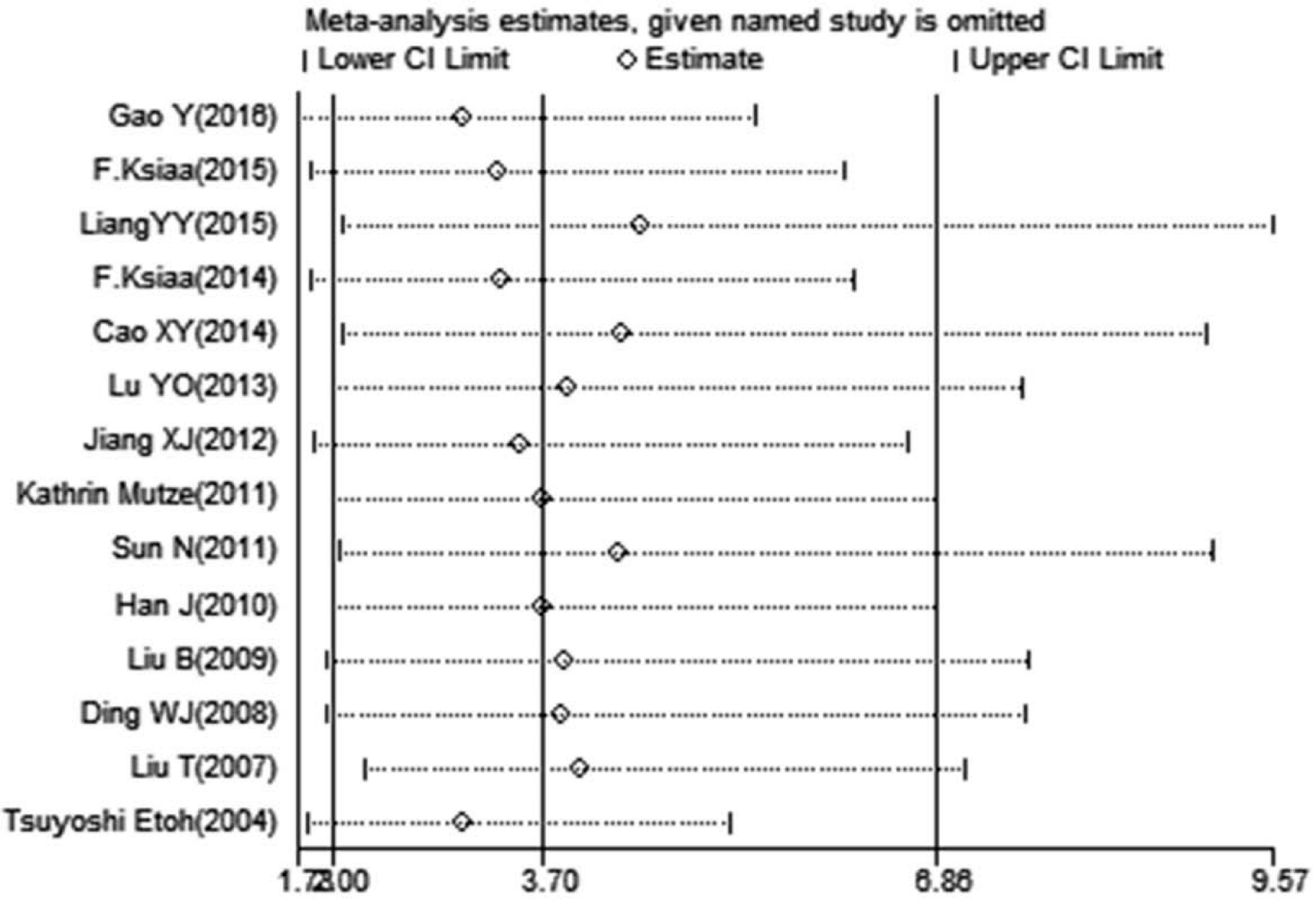
Sensitivity analysis

Publication bias existed in our meta-analysis (Begg's test, *P* = 0.040; Egger's test, *P* = 0.047). The Funnel Plot graphics showed the publication bias (Figure 3). The sensitivity analysis indicated that before and after the deletion of each study, the results were stable, demonstrating the stability of the meta-analysis (Figure 4).

### Association between DNMT1 expression and the risk, clinicopathological and prognostic significance of GC using informatics analysis

First, we integrated the raw data from TCGA database to investigate the association between DNMT1 expression and the risk of GC. The results showed DNMT1 expression was higher in GC than that in normal tissues (*P* < 0.0001). However, the abnormal expression of DNMT1 had no significant relations with some clinicopathological parameters, such as gender, age, TNM stage, histological type and prognosis (Table 2).

**Table 2:** Association between DNMT1 expression and the pathologic parameters and prognosis of GC from TCGA database

	*N*	*P*
Gender		
male	243	0.412
female	137	
Age		
> 60	255	0.856
≤ 60	125	
TNM stage		
I-II	167	0.368
III-IV	190	
histological type		
intestinal	168	0.424
diffuse	63	
prognosis		
dead	150	0.194
alive	230	

**Figure 5:**
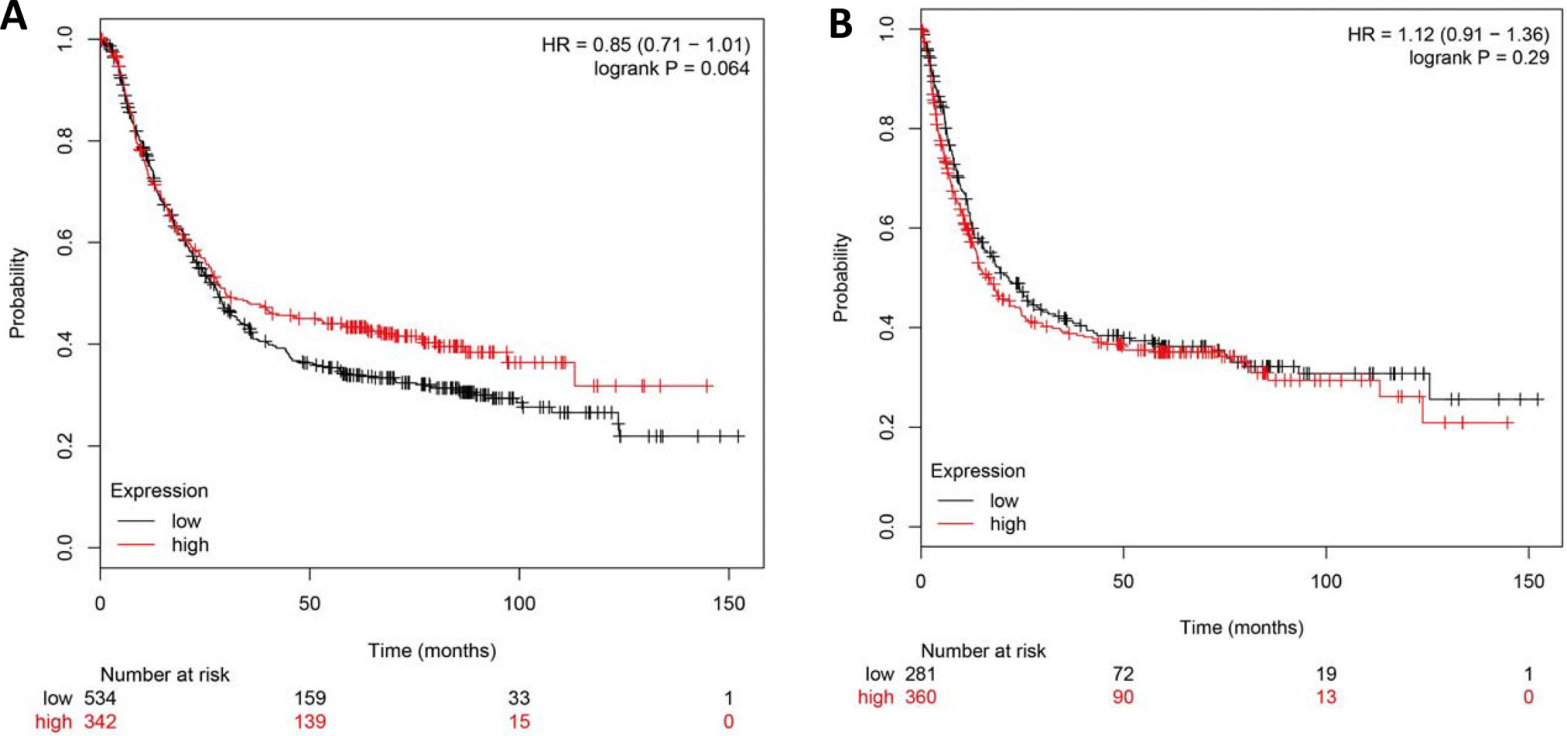

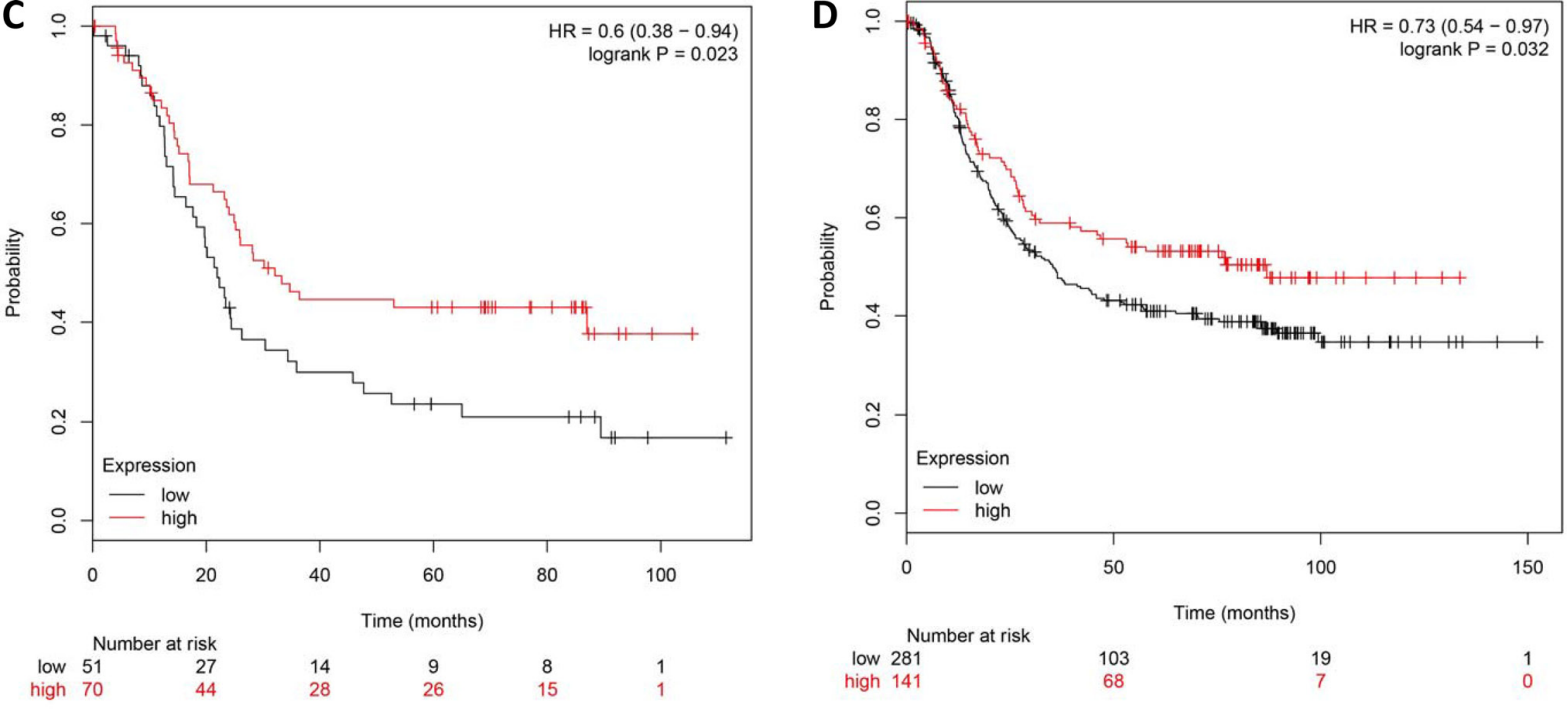

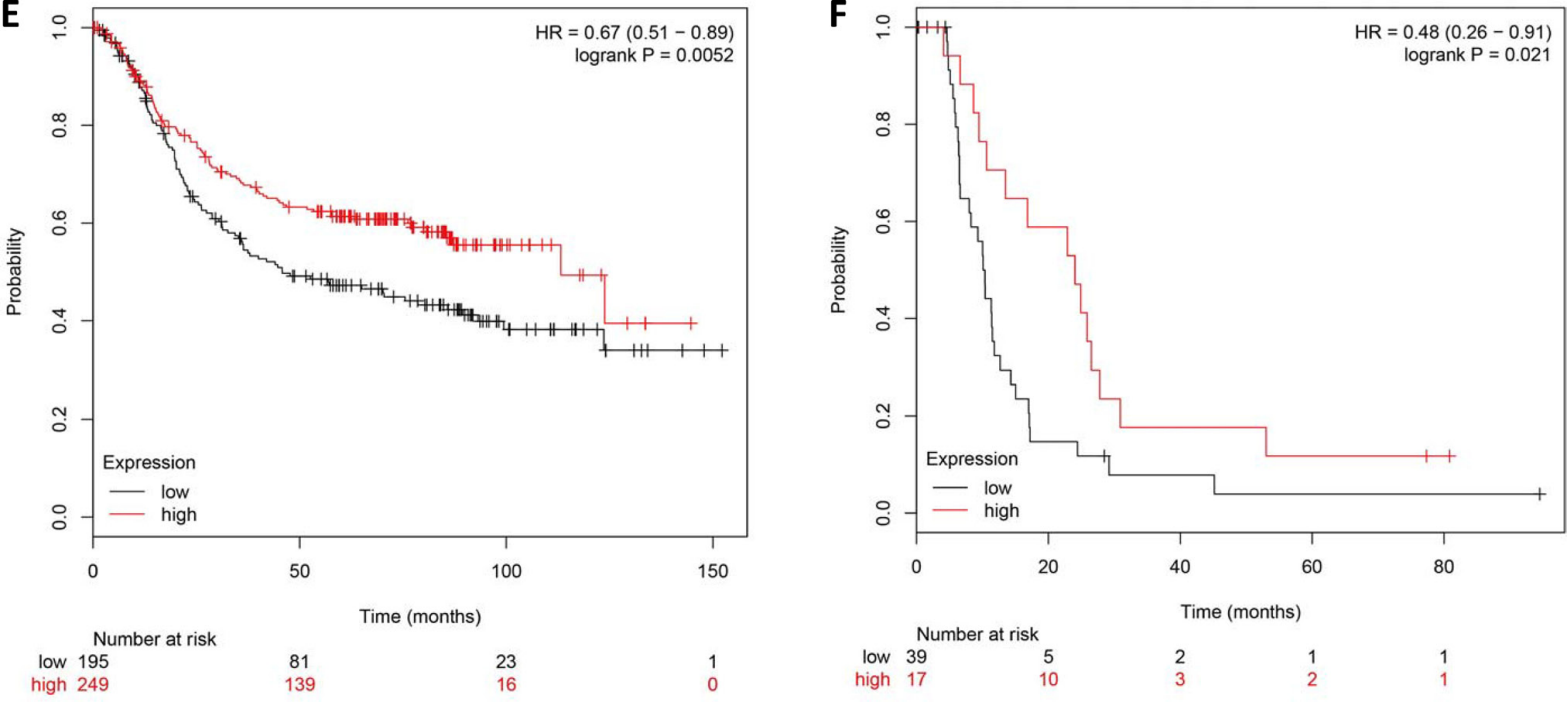

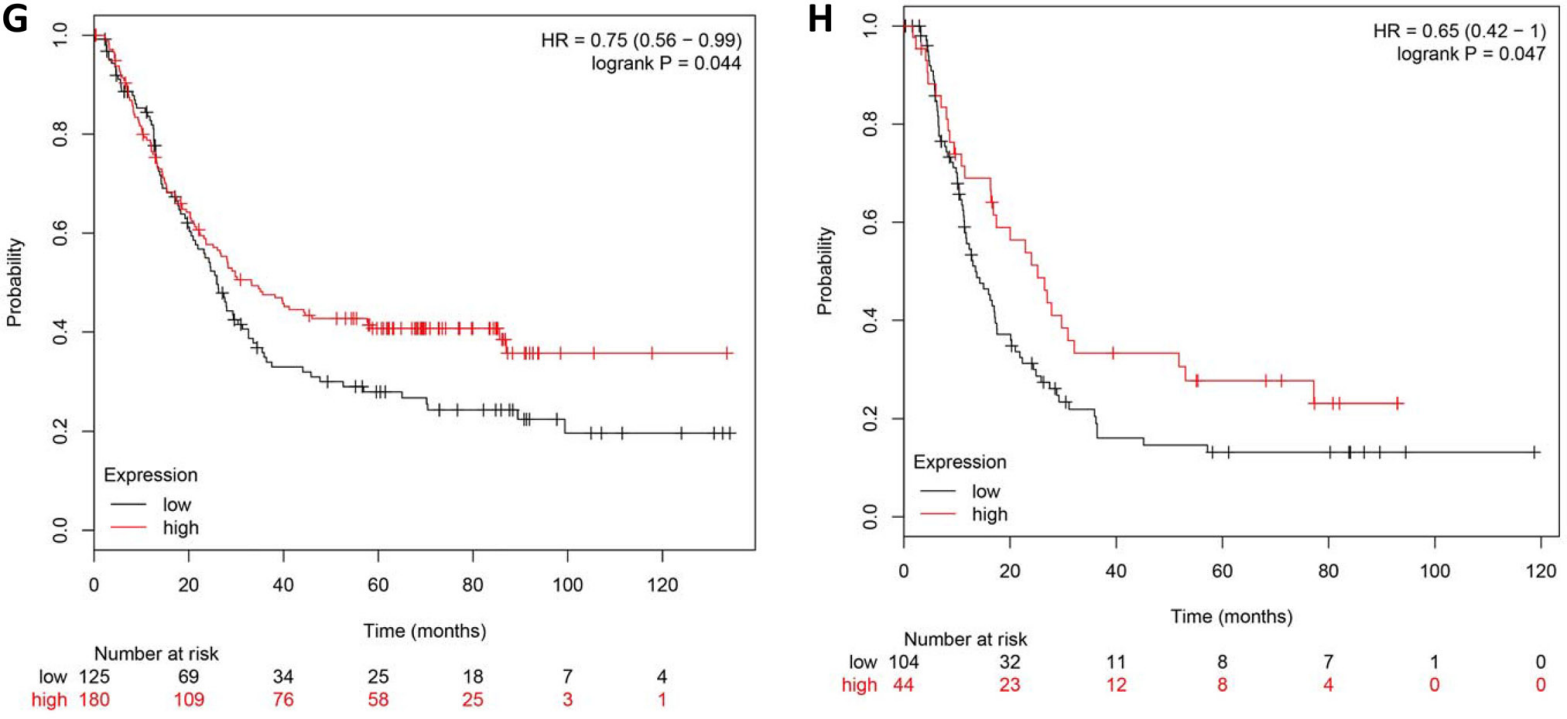

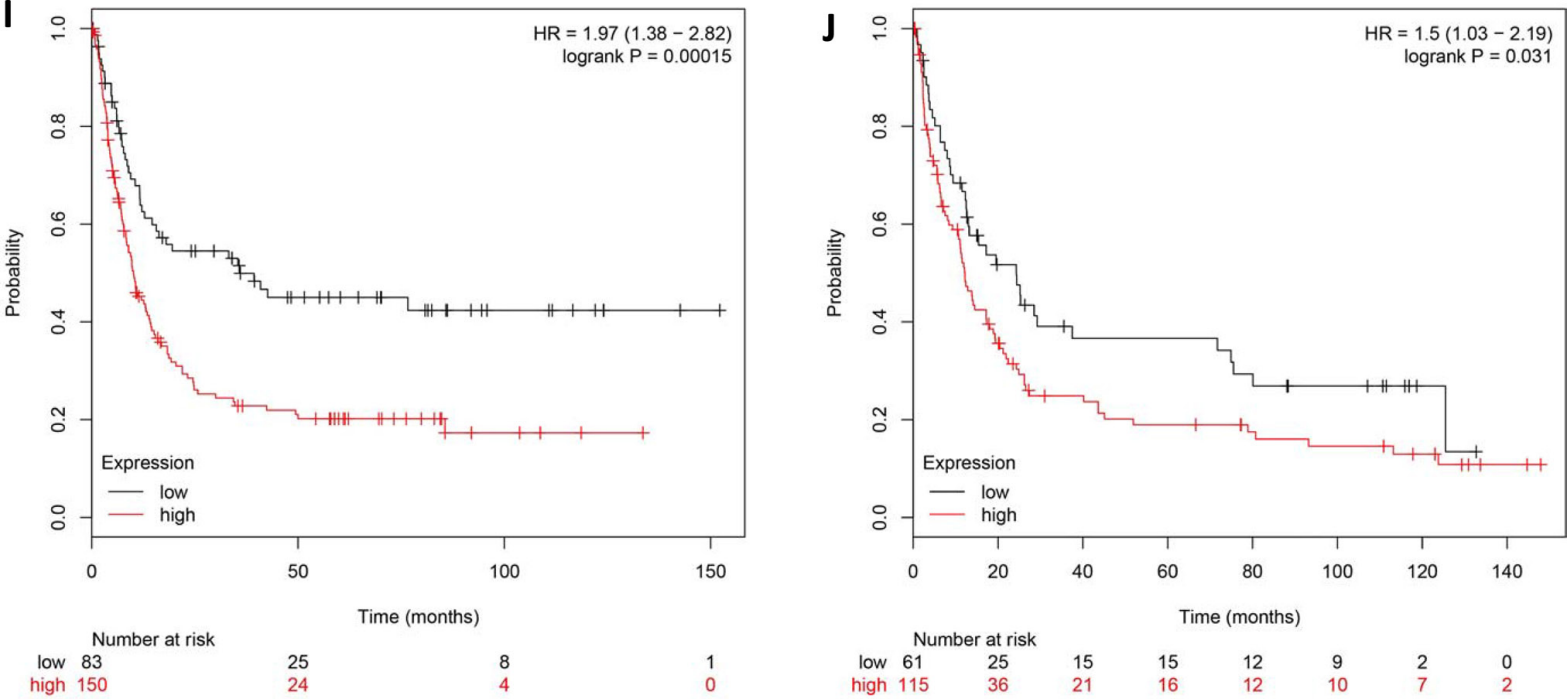

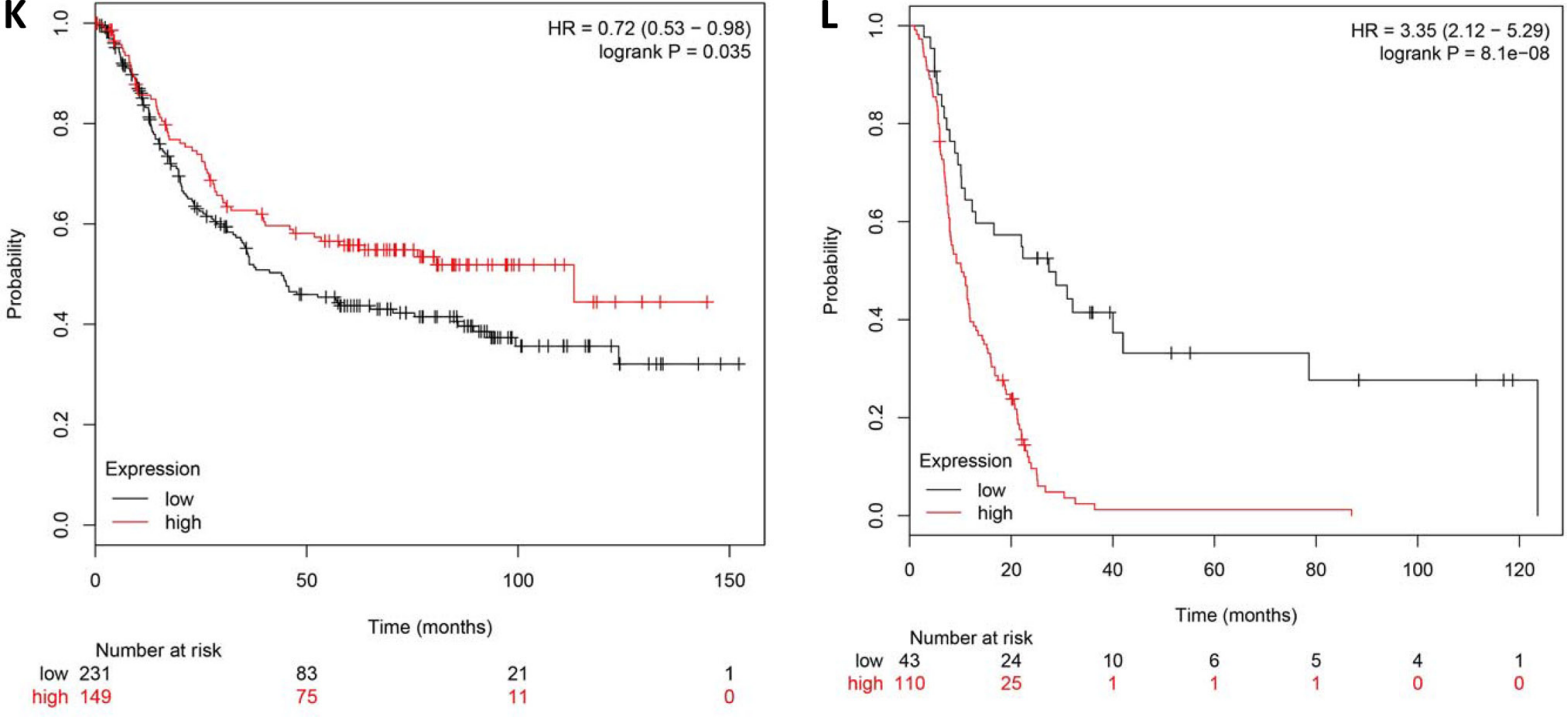

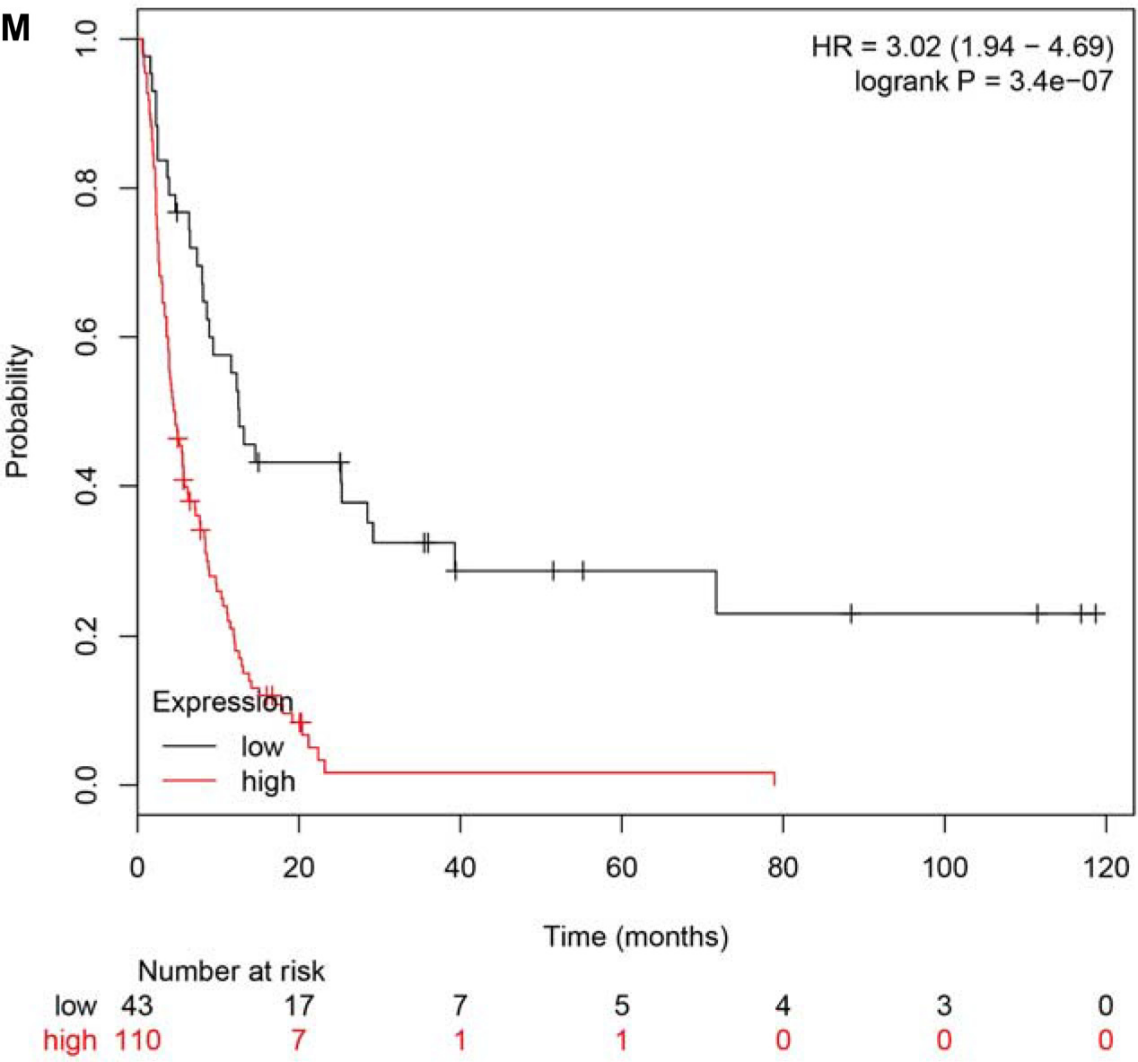
The prognostic significance of DNMT1 mRNA in the patients with gastric cancer according to the database from Kaplan Meier plotter DNMT1 mRNA expression was not related to both overall (**A**) and progression-free (**B**) survival rates of the patients with gastric cancer. DNMT1 mRNA expression has relation with OS in N2 (**C**), N1-3 (**D**), M0 (**E**), M1 (**F**), Stage 3 (**G**), Stage 4 (**H**), Surgery alone (**K**), 5-Fu (**L**), 5-Fu (**M**), and with PFS in Her2+ (**I**) and Her2- (**J**).

Second, using the Kaplan-Meier plotter, we found that DNMT1 mRNA expression was not associated with the overall survival (OS) or progression-free survival (PFS) in all GC patients (*P* > 0.05, Figure 5A, 5B). However, DNMT1 overexpression in patients with lymph node metastasis (N2, N1-3) and distant metastasis (M0, M1) showed a long overall survival time (*P* < 0.05, Figure 5C–5F), and those in stage III and stage IV also showed a good outcome for OS (*P* < 0.05, Figure 5G, 5H). For DNMT1 negative patients, the PFS with Her2+ or Her2- was better than those in positive ones (*P* < 0.05, Figure 5I, 5J). In addition, surgery alone was effective for the OS of patients with DNMT1 up-regulation (*P* = 0.035, Figure 5K), but 5-Fu were useful for the patients with DNMT1 negative expression (*P* < 0.05, Figure 5L, 5M). Therefore, DNMT1 overexpression might be considered as a reference marker to select clinical therapeutic regimen.

## DISCUSSION

DNMT1, as the most important enzyme of maintaining DNA methylation, widely exists in many kinds of tumors. However, the relationship between DNMT1 overexpression and the risk, clinicopathological parameters and prognostic of GC is indefinite. In the present study, we used a meta-analysis to summarize literature review on the correlation between DNMT1 expression and GC risk, as well as its clinical pathological parameters. On account of a limited data with the GC prognosis, we made a bioinformatic analysis of TCGA database to explore the association of DNMT1 overexpression with clinical outcomes of GC patients.

As the results showed in our meta-analysis, DNMT1 expression in GC was much higher than that in non-cancerous, which suggested that DNMT1 could be a good biomarker for diagnosis of GC. Furthermore, we compared the difference of DNMT1 expression between normal, para-cancerous, dysplasia tissues and GC tissues, respectively. We could speculate from the results above that with the increase of the severity of the gastric disease, DNMT1 protein expression increased at the same time. The results were basically consistent with the literatures we included.

We also found that DNMT1 overexpression was related to gender, differentiation and TNM stage in GC. DNMT1 preferentially expressed in men than women according to seven articles of our meta-analysis, and two of them got the same results [17, 20]. It's probably because that the mean age of the patients in these two reports were older than others. The expression of DNMT1 in patients with poor differentiation was higher than well and moderately differentiation, which was consistent with Liang [24], Sun [26] and Etoh [21]. Moreover, the up-regulated expression of DNMT1 was very common in III-IV stage when compared to that in I-II stage, although only one article revealed such difference [26]. However, as we can see, this overexpression of DNMT1 was not associated with age, tumor location, Lauren's classification, depth of invasion, lymph lode metastasis and vascular metastasis. Majority researches were similar to the results above, whereas some studies verified that patients with lymph node metastasis may increase DNMT1 expression.

Furtherly, we used bioinformatic databases to investigate the role of DNMT1 expression in the progression of GC. Using TCGA database, we found that the expression of DNMT1 was highly correlated with the risk of GC, as the statistically significant for the difference of DNMT1 expression between the cancer group and the normal group. We also analyzed the relationship between the expression of DNMT1 and parts of pathological parameters of GC through TCGA, although the results were not completely the same as our meta-analysis, there were still some similarities. On the basis of TCGA results, no differential expression of DNMT1 has been found between age (take 60 years as the line) and histological type, which was in accordance with our consequence. As for the difference between TCGA and our meta results in gender and TNM stage, this might because the patients were mainly from hospital, and most of them were Chinese.

Analysis on databases of Kaplan-Meier plotter showed that DNMT1 expression has no relation with prognosis. However, we found a relationship between DNMT1 expression and prognostic significance with clinical parameters, such as lymph node metastasis, distant metastasis and TNM stage. Besides, surgery alone had effectivity for patients with DNMT1 positive expression, but 5-Fu or other adjuvant was helpful for those with DNMT1 negative expression. Mutze K [30] also reported that GC patients with low DNMT1 expression would have a good outcome after 5-Fu based neoadjuvant chemotherapy. These results indicated that DNMT1 can serve as a biomarker for clinical therapeutic regimen.

There are several limitations should be noted in the study. First, patient populations were limited, as patients mainly from Asian, especially Chinese. Additionally, the patients basically came from the hospital, which also caused the patients bias. Second, the potential publication bias stems from published results being predominantly positive. Third, we mainly included the studies that the method of immunohistochemistry with full-texts, however there were still some unpublished personal data or meeting abstracts unavailable. Fourth, this limited sample size may affect the power to detect the associations in some articles.

In conclusion, we demonstrated the DNMT1 abnormal overexpression might be employed as a good potential marker for prediction of GC, and related with clinicopathological parameters, as well as the prognosis of GC. Further large-scale and well-designed studies are still needed to confirm the results of our meta and bioinformatic analysis.

## MATERIALS AND METHODS

### Identification of eligible studies and selection criteria

We performed a publication search using PubMed, Web of Science, BIOSIS, and CNKI updated on Jun 5th, 2017, and only the human studies were searched. The following search terms were used: (DNMT1 OR MCMT) AND (gastric OR stomach) AND (cancer OR carcinoma OR adenocarcinoma)). Searching was done without restriction on language or publication years.

**Figure 6:**
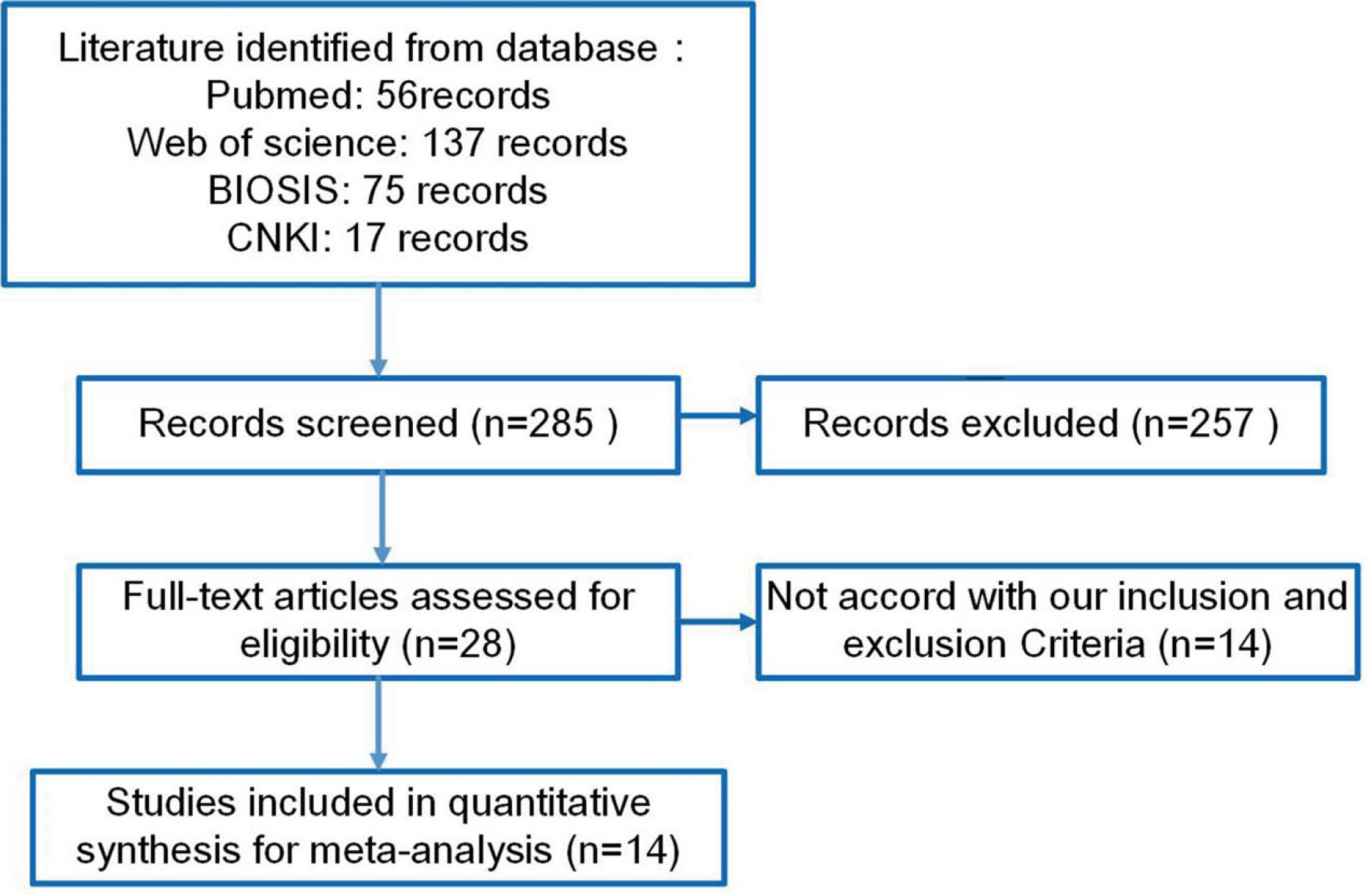
Flow chart of the selection process in this meta-analysis

Inclusion criteria for studies: (1) relevant articles to observe the DNMT1 protein expression in GC (2) papers to compare the DNMT1 expression with risk and clinicopathologic significance of GC by immunohistochemistry. Exclusion criteria included: (1) abstract, comment, review and meeting; (2) duplication studies of previous reports; (3) Western blot, RT-PCR, or other detection method for DNMT1 expression; (4) lack of sufficient information. Finally, 15 articles were selected for our meta-analysis (Figure 6).

### Data extraction

Based on the inclusion criteria, two independent reviewers (TM Ma and LP Sun) extracted information from all eligible publications. We summarized the information and statistical data that extracted from full-text format. The following information was extracted in each study: name of first author, year of publication, country, ethnicity, antibody company, numbers of cases and controls, risk to cancer, aggress and outcome. Any disagreement was solved through discussion until the two reviewers reached a consensus (Table 1).

### Quality score assessment

Two reviewers (TM Ma and LP Sun) independently assessed the quality of the 15 studies by using Newcastle Ottawa Scale (NOS). The scale contains three parts: sample selection, comparability and exposure outcome. Based on the quality score criteria, the higher scores indicated, the better quality 10 points is our top score, and more than 5 points was accepted in our meta-analysis.

### Bioinformatic analysis

We downloaded 443 patients’ data from stomach database in Cancer Genome Atlas (TCGA) by TCGA-assembler with R software. Among them, 408 patients were with the normal stomach or gastric cancer, and 380 patients had clinicopathologic significance and prognostic information. We just picked out 408 cases to evaluate the GC risk, and 380 cases to analyze the clinicopathologic significance and prognostic of GC. Furthermore, the prognostic significance of DNMT1 mRNA was analyzed using Kaplan-Meier plotter.

### Statistics analysis

Statistical analyses were carried out by Review Manager vision 5.3 (The Cochrane Collaboration, Nordic Cochrane Centre Copenhagen, Denmark).The relationship between DNMT1 expression and cancer risk, different clinicopathologic characteristics were assessed by odds ratios (OR) with 95% confidence intervals (CI). Statistical significance of the pooled OR was determined by *Z* test, and it was meaningful when *P*-value < 0.05. Heterogeneity effect was quantified by the Chisquare-based *Q* test and the I^2^ test. An I^2^ < 50% or *P*-value > 0.10, indicated no significant heterogeneity, and a fixed-effects model (Mantel-Haenszel method) was used. Otherwise, the random-effects model (DerSimonian and Laird method) was used. We explored the possible source of statistical heterogeneity by subgroup analysis according to the ethnicity of patients, and sensitivity analysis. Publication bias and sensitivity analysis were calculated by State vision 11.0 (State corporation, College Station, TX, USA). Publication bias was estimated by Begg's test and Egger's test to assess funnel plot asymmetry. Data from TCGA database was dealt by *t*-test using SPSS vision 22.0 (IBM SPSS statistics, Armonk, NY, USA), and *P*-value < 0.05 was considered as statistically significant.
